# Human papillomavirus infection and risk factors in a cohort of Tuscan women aged 18-24: results at recruitment

**DOI:** 10.1186/1471-2334-10-157

**Published:** 2010-06-07

**Authors:** Massimo Confortini, Francesca Carozzi, Marco Zappa, Leonardo Ventura, Anna Iossa, Paola Cariaggi, Livia Brandigi, Mario Franchini, Francesco Mirri, Paolo Viacava, Aurora Scarfantoni, Daniela Bazzanti, Cristina Sani

**Affiliations:** 1 Analytical and Biomolecular Cytology Unit, Cancer Prevention and Research Institute, ISPO, Via Cosimo il Vecchio 2, Florence, Italy; 2 Clinical and Descriptive Epidemiology Unit, Cancer Prevention and Research Institute, ISPO, Via di S. Salvi, Florence, Italy; 3 Screening Unit, Cancer Prevention and Research Institute, ISPO, viale A. Volta 171, Florence, Italy; 4 Cytopathology Unit, Cancer Prevention and Research Institute, ISPO, viale A. Volta 171, Florence, Italy; 5 Obstetrics and Gynecology Unit, Santa Maria Annunziata Hospital, via dell'Antella, Florence, Italy; 6 Departimental Section of Cytohistological for Oncological Screening, via Curtatone 54, Arezzo, Italy; 7 Pathological Anatomy Department, Ospedale Unico della Versilia, via Aurelia 335, Lido di Camaiore, Lucca, Italy; 8 Sanitary District of Valdarno, via Curtatone 54, Arezzo, Italy

## Abstract

**Background:**

There is conclusive evidence that human papillomavirus (HPV) infections of the cervix are a necessary cause of cervical cancer. In Italy there are consistent data of HPV prevalence in women aged 25 - 64 years, but there is limited data for younger women. The objective of this on-going 3-year prospective cohort study is to investigate the prevalence, acquisition, clearance and persistence of HPV infections in young Tuscan women and the risk factors correlated with such events.

**Methods:**

One thousand and sixty-six women aged between 18 and 24 years were enrolled and received an initial HPV test. They were asked to return to the clinic over the study period for further tests every 12 months, if their HPV HR result was negative, or every 6 months, if positive. Additionally, women with an HPV positive result were given a cytological examination and if the cytological diagnosis was ASC-US or more severe, only women with HPV HR, were referred for colposcopy.

**Results:**

We present here data for the enrolment phase of the study. At baseline, within the study sample, just under 30% of women were infected by HPV and 19.3% of women were infected with oncogenic types. A relationship was highlighted between HPV infection, number of sexual partners (in particularly in the last 3 years) and the lifetime number of partner's partners. Condom use showed a slight protective effect in univariate analysis but these data were not statistically significant in multivariate analysis. The association between HPV infection and demographic and behavioural variables were tested by crude odds ratio (OR). Multivariate logistic regression was applied to compute the adjusted odds ratios.

**Conclusions:**

The prevalence of oncogenic HPV types was high in young Tuscan women. The 3-year follow-up of this cohort may provide a better understanding of the processes of acquisition, clearance and persistence of infection and the correlated risk factors.

## Background

Cervical cancer is the second most common cancer in women worldwide and knowledge regarding its cause and pathogenesis is rapidly expanding. Although there is conclusive evidence that infection of the cervix by some types of human papillomavirus (HPV) is a necessary cause of cervical cancer [1,2] the discrepancy between the high frequency of HPV infections in young, sexually active women and the relatively low occurrence of cervical lesions in the same population suggests that HPV is not a sufficient cause for cervical neoplasia [3]. There is evidence that most HPV infections are transient and only women who harbour a persistent HPV infection are likely to develop a cervical lesion [4,5].

The accumulated evidence that high risk types (HPV HR) are a necessary cause of cervical cancer has led to the design of prophylactic vaccines. The currently licensed vaccines are Gardasil (Merck Pharmaceuticals) and Cervarix (GlaxoSmithKline) and protect against infection by HPV types 6, 11, 16 & 18 and types 16 & 18, respectively. The vaccines, both of which have been approved for girls and young women, are designed to prevent 90% of all genital warts and 70% of all cervical cancers (Gardasil) and 70% of all cervical cancers (Cervarix). Studies have shown that types 6 & 11 are responsible for 90% of genital warts and types 16 & 18 for 70% of cervical cancers. In Italy, there are consistent HPV prevalence data based on NTCC studies for women aged 25 - 64 years [6,7] but there is a paucity of data for younger women [8]. The objective of this prospective cohort study is to investigate the prevalence, acquisition, clearance and persistence of HPV types in unvaccinated women aged 18 - 24 years and the risk factors correlated with such events. For this age group, that precedes the onset of screening, there are very limited data of HPV prevalence in Italy. This on-going 3-year prospective cohort study was initiated in June 2007. Here we report the baseline results.

## Methods

### Study population

The study sample was recruited from three areas of Tuscany (Florence, Valdarno Aretino and Viareggio - respectively, urban, rural, and coastal areas) each with good experience in delivering population-based cervical cancer screening programmes which, following Italian national guidelines [9], include a triennial recall of all women aged 25 - 64 years for a Pap test.

With the intention to enrol at least 1000 women and on the assumption of a 10% and 15% compliance to invitation, approximately 8000 women aged 18 - 24 years were randomly selected from Tuscan population registries, and were sent a letter with a request that they participate by calling and fixing an appointment. In cases of no response, a reminder was sent.

### Protocol

**Figure 1:**
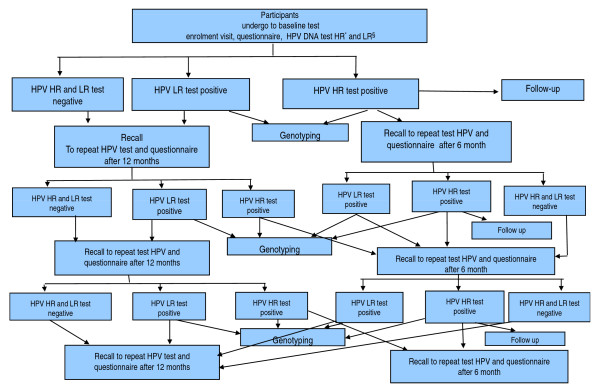
**Study profile. A ) Enrolment**. * HPV HR= Human papillomavirus High Risk § HPV LR= Human papillomavirus Low Risk.

The study protocol is briefly summarized in the following lines and shown graphically in Figure 1. At enrolment, women were not considered eligible, if they were not sexually active, were pregnant, had undergone hysterectomy, had been treated for CIN, had a genetic, chronic or dysmetabolic disease, were vaccinated for HPV or had an immunodeficiency disease. All eligible women received an initial HPV test. They were asked to return to the clinic for a further test at 12 months, if their HPV HR result was negative, or at 6 months, if positive. Additionally, women with an HPV HR positive result were given a cytological examination and, if the cytological diagnosis was ASC-US or more severe, were referred for a colposcopy. Women who were HPV LR positive were not referred for colposcopy whatever their cytological diagnosis. If the colposcopy result was negative women were recalled to repeat the HPV test after 6 months, but if positive, the suspicious areas identified by colposcopy were biopsied. Histology was read locally by pathologists and was not masked to cytology or HPV result. Women followed the routine protocol of treatment or follow-up according to histological results. This regime is being repeated throughout the 3-year study period.

The study was approved by the local ethics committee in each area.

### Examination

At enrolment, trained midwives explained the study aims and procedures. A written informed consent was given by each participating woman. The cervical cell samples, obtained by using a plastic Ayre's spatula and a cytobrush, were put in PreservCyt solution (ThinPrep; Cytyc Corporation, Boxborough, MA) and were used for HPV testing and for cytological examination.

Participants provided information on socio-demographic characteristics and sexual history. In a face-to-face, standardized interview participants gave information on age, race/ethnicity, education, occupation, reproductive and menstrual factors, number of pregnancies and smoking habits, and in a self-administered questionnaire at the clinic participants provided information on sexual history - age at first intercourse, age of the first partner at time of first intercourse, number of sexual partners in participant's lifetime and in the last year, lifetime number of partner's partners and use of contraceptive methods and condoms. During subsequent visits to the clinic, information on changes in smoking and sexual habits since baseline examination was requested, and endo- and ectocervical cells from the uterine cervix were collected.

### HPV Testing

The Hybrid Capture 2 assay (HC2; Digene Corporation, Gaithersburg, MD) was used for HPV testing. The two groups of probes designed to detect high-risk HPV types (16, 18, 31, 33, 35, 39, 45, 51, 52, 56, 58, 59, & 68) and low-risk types (6, 11, 42, 43, 44) were used. Four mL of PreservCyt sample was processed with the Sample Conversion Kit (Digene Corporation, Gaithersburg, MD) followed by an HC2 assay, according to the manufacturer's instructions. HC2 results were expressed in Relative Light Units (RLU) as the ratio of the specimen's light emission to that of three concurrently tested 1 pg/mL HPV DNA controls. An RLU is an (indirect) measure of the specimen's viral concentration relative to 1 pg/mL.

### Typing

All HPV HR positive samples were typed using the 'Consensus High Risk HPV genotyping' kit (Digene Corporation, Gaithersburg, USA). The test is based on the reverse hybridization principle. Denaturated biotynilated amplicons, resulting from the amplification of a part of the L1 region with GP5+/GP6+ primers, were hybridized with specific oligonucleotide probes, which were immobilized as parallel lines on membrane strips. After hybridization and stringent washing, streptavidin-conjugated alkaline phosphatase was added and bound to any previously formed biotinylated hybrid. Incubation with chromogen yielded a purple precipitate and the results could be visually interpreted. The kit allows an easy and reliable identification of 18 HPV HR types (16, 18, 26, 31, 33, 35, 39, 45, 51, 52, 53, 56, 58, 59, 66, 68, 73, & 82).The HPV HR positive samples that were negative in the PCR enzyme-linked immunoadsorbent assay were typed with cycle sequencing and analyzed on an ABI PRISM 310 DNA Genetic Analyzer (Applied Biosystems, Foster City, CA). All nucleotide sequences were analyzed and compared with HPV type sequences in GenBank using BLAST 2.0 (NCBI-NLM-NHI Bethesda, MD). A sequence was considered a match if it had a 90% nucleotide identity to an HPV sequence in GenBank.

In order to identify the HPV LR types, samples positive for HC2 probe A (specific for LR types) were amplified using specific primers for five HPV LR types (6, 11, 42, 43, and 44).The amplified products were analyzed by gel electrophoresis in a 2% agarose gel containing ethidium bromide, and observed under ultraviolet light.

HPV infections were defined by oncogenic risk ( i.e. high or low risk) and phylogenetic species (species 1, types 42 and 89; species 3, types 61, 62, 72, 81, 83 & 84; species 5, types 26, 51 & 82; species 6, types 53, 56 & 66; species 7, types 18, 39, 45, 59, 68 & 70; species 9, types 16, 31, 33, 35, 52, 58 & 67; species 10, types 6, 11 & 55).

### Cytology

Liquid-based cytology was performed using the ThinPrep system (Cytyc Corporation, Boxborough, MA ) for all women who were HPV positive [10,11]. One slide was prepared for each woman according to the manufacturer's instructions. Abnormal slides were reviewed by a panel of cytologists before they reported the results to the women. Cytology was classified according to the Bethesda 2001 Guidelines (TBS 2001) [12].

### Statistical analysis

The association between HPV infection and demographic and behavioural variables was tested by crude odds ratio (OR). Multivariate logistic regression was applied to compute the adjusted odds ratios.

All variables were analyzed in the univariate analysis, but in the multivariate analysis we adjusted the model for the statistically significant variables and those variables that, though not statistically significant, play an important biological role.

All tests were two-sided. Stata 10 software was used for analysis.

## Results

Recruitment commenced in June 2007 and was completed by June 2008 with the enrolment of 1066 women. Seven women had been excluded as they did not meet the eligibility criteria. In Florence, Valdarno Aretino and Viareggio, respectively, 542, 289, and 235 women were enrolled. The overall compliance to invitation was 15.7% with no statistically relevant difference between areas (15.4%, 16.2% and 15.8% for Florence, Valdarno Aretino and Viareggio, respectively). The compliance by age is shown in Table 1. The mean age of the participants at enrolment was 21.6 years (SD = 1.87).

**Table 1:** Compliance and HPV HR and LR positivity by age at baseline.

	Participants at baseline		HPV HR positives	HPV LR positives
	
Age in years	N°	(%)	N°	N°
18	62	(5,82)	9	5
19	130	(12,20)	17	11
20	160	(15,01)	35	20
21	148	(13,88)	27	11
22	183	(17,16)	40	14
23	178	(16,70)	32	18
24	205	(19,23)	46	28

### Prevalence at enrolment

**Figure 2:**
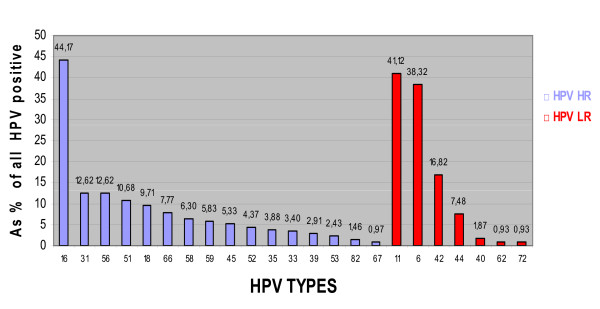
**Distribution of HPV HR and LR types**.

Of the study sample, 206 (19.32%) women were HPV HR positive and 107 (10.03%) were HPV LR positive. Table 1 shows the HR/LR status by age. Infections with HPV HR types alone were detected in 146 (13.70%) women, infections with HPV LR types alone were detected in 47 (4.41%)women and co-infections by HPV HR and LR types were detected in 60 (5.63%) women. HPV 16, the most prevalent type (8.53%), was followed by types HPV 31 and HPV 56 (2.44%), HPV 51 (2.06%) and HPV 18 (1.88%). The HPV LR vaccine types, 6 & 11, were detected in 3.47% and 4.12% of women, respectively. Co-infections with two or more HPV HR types were revealed in 46 (4.32%) women. The prevalence of HPV HR vaccine types, whether single or in co-infection, was 10.41%. Concomitant HPV 16 & 18 infections were present in 5 women only (0.47%). The distribution of the 23 HPV types identified, as a percentage of the total HPV positive women (low and high risk), is shown in Figure 2.

**Figure 3:**
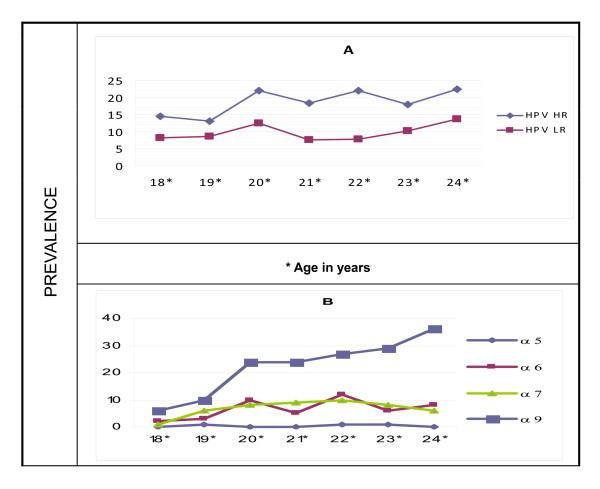
**Prevalence of cervical HPV infection oncogenic risk (A) and phylogenic species (B) by age**.

Figure 3 shows the prevalence by age for cervical HPV infection by oncogenic risk (A) and phylogenic species (B) (the types in a species having common biological and pathological properties).

### Cytology

The association between HPV infection and the presence of cervical cytological abnormalities was examined in all HPV positive participants. Table 2 shows the association between cytological outcomes and classes of viral oncogenic risk. The only HSIL diagnosis was associated with an HPV HR single infection. All cytological diagnoses ASCUS +, in patients HPV LR positive, were associated with HPV types 6 & 11.

**Table 2:** Association between cytological outcomes and class of viral oncogenic risk.

	CLASS OF RISK					
	
	LOW RISK N° (%)	SINGLE HIGH RISK N° (%)	LOW/HIGH RISK N° (%)	MULTIPLE HIGH RISK N° (%)	NOT TYPING N° (%)	TOTAL N° (%)
CYTOLOGY						
NEGATIVE	32 (72.73)	80 (79.21)	36 (60.00)	33 (71.74)	2 (100)	183 (72.33)
ASC-US	7 (15.91)	12 (11.88)	11 (18.33)	7 (15.22)	0	37 (14.62)
LSIL	4 (9.09)	8 (7.92)	11 (18.33)	6 (13.04)	0	29 (11.46)
HSIL	0	1 (0.99)	0	0	0	1 (0.40)
ASC-H	0	0	2 (3.34)	0	0	2 (0.79)
NOT ESTIMABLE	1 (2.27)	0	0	0	0	1 (0.40)

TOTAL	44	101	60	46	2	253

Compliance to colposcopy was 82.76% (48/58): 18 had negative results, 24 had histological diagnoses lower than CIN2, 5 had a histological diagnosis CIN 2 and 1 had a histological diagnosis CIN 3.

### Univariate analysis

Table 3 shows the univariate analysis of participants' socio-behavioural characteristics and HPV infection status. The median ages for the first sexual intercourse and of the first partner were 17 and 19 years, respectively. Some 64.2% of participants (719/1066) reported to have had more than one partner and 11.1% of participants reported more than four partners (3.3% missing). A lower age at first intercourse, a greater number of sexual partners (in lifetime and in the last three years) and a greater lifetime number of partner's partners were strongly associated with prevalence of HPV infection.

**Table 3:** HPV prevalence by socio-behavioral characteristics: univariate analysis.

Characteristics	**No**.	HPV positive n (%)	Crude OR (95% CI)	P
**Condom use**				0.01
Never used	262	75 (28.6)	1.00	
Used sometimes	305	83 (27.2)	0.93 (0.64-1.34)	
Used most of the time	245	52 (21.2)	0.67 (0.44-1.00)	
Always used	238	40 (16.8)	0.50 (0.33-0.77)	
Missing	16	3 (18.8)	0.57 (0.16-2.07)	
**Age at the first intercourse**				0.01
≤ 17 years old	697	182 (26.1)	1.00	
> 17 years old	369	71 (19.2)	0.67 (0.49-0.91)	
**Number of partners the last three years**				<0.0001
1	532	52 (9.8)	1.00	
2 - 4	421	141 (33.5)	4.65 (3.27-6.60)	
≥ 5	86	49 (56.9)	12.22 (7.31-20.43)	
Missing	27	11 (40.7)	6.34 (2.79-14.39)	
**Total number of partners**				<0.0001
1	347	26 (7.5)	1.00	
2 - 4	474	116 (24.5)	4.00 (2.54-6.28)	
≥ 5	210	97 (46.2)	10.59 (6.53-17.17)	
Missing	35	14 (40.0)	8.23 (3.75-18.05)	
**Lifetime number of partner's partners**				<0.0001
1	263	12 (4.5)	1.00	
2 - 4	218	41 (18.8)	4.84 (2.47-9.48)	
≥ 5	119	41 (34.4)	10.99 (5.50-21.95)	
Missing	466	159 (34.1)	10.83 (5.88-19.93)	
**Smoking**				<0.0001
No	639	116 (18.1)	1.00	
Yes	422	137 (32.5)	2.16 (1.62-2.88)	
Missing	5	0 (0.0)		
**No. of cigarettes**				<0.0001
0	639	116 (18.1)	1.00	
1 - 10	337	101 (29.9)	1.92 (1.41-2.62)	
> 10	73	35 (47.9)	4.15 (2.51-6.85)	
Missing	17	1 (5.8)	0.28 (0.03-2.14)	
**Oral contraceptives (current use)**				0.83
No	577	141 (24.5)	1.00	
Yes	481	110 (22.9)	0.91 (0.68-1.21)	
Missing	8	2 (25.0)	1.03 (0.20-5.16)	
**Pregnancies prior**				0.04
Yes	78	26 (33.3)	1.00	
No	974	226 (23.2)	0.60 (0.36-0.99)	
Missing	14	1 (7.1)	0.15 (0.01-1.24)	
**Genital disease**				0.002
Yes	285	89 (31.2)	1.00	
No	773	162 (20.9)	0.58 (0.43-0.79)	
Missing	8	2 (25.0)	0.73 (0.14-3.70)	
**Partner's genital disease**				0.27
Yes	54	16 (29.6)	1.00	
No	1000	236 (23.6)	0.73 (0.40-1.33)	
Missing	12	1 (8.3)	0.21 (0.02-1.81)	
**Education**				0.07
University	94	22 (23.4)	1.00	
High school	815	181 (22.2)	0.93 (0.56-1.54)	
Middle school	132	41 (31.06)	1.47 (0.80-2.69)	
Missing	25	9 (36.0)	1.84 (0.71-4.74)	

**Total**	1066	253 (23.7)		

Some 45.1% of participants reported no current use of oral contraception (8 missing). The current use of oral contraceptives in the univariate analysis showed no effect on prevalence of infection.

Four hundred and twenty two women were smokers (39.2%, 5 missing). Smoking increased the risk of infection (OR = 2.16, 95% CI, 1.62-2.88).

Women reporting an occasional use of condoms (28.6%) had a similar risk to those who never used them (24.6%) (OR = 0.93, 95% CI, 0.64-1.34), whereas an increasing protection was observed for women who used condoms most of the time (23.0%) (OR = 0.67 95% CI, 0 4.4-1.00) or always (22.3%) (OR = 0.50, 95% CI, 0.33-0.77).

No previous pregnancies showed a protective effect (OR = 0.6, 95% CI, 0.36 -0.99) as did not reporting previous genital disease (OR = 0.58, 95% CI, 0.4-0.8). No partner's genital disease showed a not statistically significant protective effect (OR = 0.73, 95%CI, 0.4-1.3). A lower level of education was associated with a not statistically significant increased risk of infection.

### Multivariate analysis

Table 4 shows the results of the multivariate analysis, which included all the statistically significant variables (plus age) of the univariate model. In this analysis, the effects of the number of sexual partners in the last three years and the lifetime number of partner's partners both remained statistically significant. In comparison with women reporting a partner who had had only one lifetime partner (the study participant) there was more than a three-fold increase in risk for those whose partner had between 2 and 4 lifetime partners (OR = 3.2, 95% CI, 1.60-6.43). The increase was more than five-fold when the women's partner had 5 or more lifetime partners (OR = 5.45, 95% CI, 2.62-11.33). In comparison with non-smokers, women smoking more than ten cigarettes per day had twice the risk of infection (OR = 2.22, 95% CI, 1.25-3.93).

**Table 4:** HPV infection by sociobehavioral characteristics: multivariate analysis.

Characteristics	Adjusted OR (95% CI)
**Condom use**	
Never used	1.00
Used sometimes	0.86 (0.57-1.31)
Used most of the time	0.72 (0.45-1.15)
Always used	0.84 (0.51-1.37)
Missing	0.66 (0.16-2.78)
**Age**	
Continuously variable	1.08 (0.98-1.19)
**Age at the first intercourse**	
Continuously variable	0.95 (0.86-1.06)
**Number of partners the last three years**	
1	1.00
2 - 4	3.42 (2.34-4.99)
≥ 5	6.83 (3.88-12.02)
Missing	3.82 (1.54-9.50)
**Lifetime number of partner's partners**	
1	1.00
2 - 4	3.20 (1.60-6.43)
≥ 5	5.45 (2.62-11.33)
Missing	5.42 (2.87-10.25)
**No. of cigarettes**	
0	1.00
1 - 10	1.21 (0.86-1.72)
> 10	2.22 (1.25-3.93)
Missing	0.32 (0.04-2.84)
**Pregnancies prior**	
Yes	1.00
No	0.74 (0.41-1.33)
Missing	0.17 (0.02-1.59)
**Genital disease**	
Yes	1.00
No	0.80 (0.57-1.14)
Missing	2.34 (0.34-16.2)

After adjustment of the other covariates condom use, no prior pregnancies, no previous genital disease and greater age at first intercourse showed a very small and not statistically significant protection.

## Discussion and Conclusions

This paper reports the baseline results at enrolment for an on-going study.

The compliance to the invitation (15%) was low in comparison with the compliance to invitations for the cervical screening programs for women aged 25 - 64 years in the same area (approximately 40%). It was particularly low for women aged 18 and 19 years.The difference may be due, in part, to the lower age of the study population and their being unaccustomed to gynaecological examination, and in part, to the test offered (the HPV test) which was still little known in Italy when the study began. However, the participation rate is very similar to that obtained in similar studies conducted in Italy [8].

Nevertheless, the participating women do not seem to differ from the general population in terms of age at first intercourse and the number of partners. In fact these characteristics were similar to those found in other Italian surveys. Ammatuna et al. in a study of 1000 Sicilian women reported a mean age of 21.6, a mean age of first sexual intercourse of 17.4, and mean number of lifetime partners of 2.22 [8].

Our baseline result that almost 30% of sexually active women aged 18 - 24 years were infected by HPV is comparable with the range (19.7% - 39%) reported worldwide for women in the same age group [13,14]. Infection with oncogenic HPV types was found in almost 20% of our sample (19.32%). This prevalence was higher than that found in NTCC studies in women aged 25 - 29 (14.25%), confirming that the prevalence of infection is higher in younger women [18-24] and tends to decrease with age [6,7,15].

Cervical infection with multiple HPV types was relatively common among Italian cohort participants, [16,17]. Goodman et al assessed that the risk of acquiring some HPV HR types was enhanced among women with coexisting cervical HPV infections [18]. Other investigators who have examined the dynamics of cervical HPV infection in the presence of other types reported that the concurrent acquisition of multiple HPV types exceeded that expected by chance [19,20] although only Mendez et al found that type specific HPV acquisition was dependent on a cervical infection with another type. Rousseau et al [21] found that HPV acquisition was more likely among women with another HPV type at study entry and that certain co-infections (e.g. HPV 16 and 52) occurred with a significant frequency. These observations suggest common exposures and common routes of transmission, although alternative explanations, such as biological interaction between various HPV types, cannot be excluded [20].

HPV 16 (8.53%) and HPV 18 (1.88%) were present in 10.41% of the study sample. This prevalence of vaccine types would suggest that the young population being studied, especially the 18 & 19 year-olds, is an appropriate target for the prophylactic vaccination. Amongst 18 year-olds the prevalence of HPV 16 was 1.94% (4/206) and of HPV 18 was 0% (0/206), whereas amongst 19 year-olds the prevalence of HPV 16 was 2.43% (5/206) and of HPV 18 was 0.97% (2/206).

Our baseline results confirm the roles of the number of sexual partners (in particularly in the last 3 years) and of the lifetime number of partner's partners [22] as determinants of HPV infection. Both these variables act as independent risk factors in the multivariate model. In fact, in comparison with women reporting one partner, the risk of infection increased more than 6 times and more than 5 times for women reporting to have had more than 5 partners and for those reporting a current partner having had more than 5 lifetime partners, respectively. These figures are consistent with all types of HPV infection as well as for HPV HR infection (data not shown). This finding is congruent with previous studies [23-25] in which the prevalence of HPV among women who had had two or more sexual partners was double that in women who had had only one partner.

At baseline smoking habits act as an independent factor of increased acquisition of HPV infections in both the univariate or multivariate models. This association has been observed in the pooled analysis of the International Agency for Research on smoking and human papillomavirus infection where the risk of being HPV positive increased with smoking intensity, after allowing for lifetime number of sexual partners [26].

The use of condoms has an slight protective effect in the univariate analysis, but is not statistically significant in the multivariate analysis, suggesting that use of condoms is associated to low risk sexual behaviours. In fact, our data shows that women who said they frequently use condoms, also indicated that they have had a low number of sexual partners.

This point is heavily debated and has important consequences. Many previous papers, in which a retrospective approach has been adopted (as in this study) [14,27-31], have shown similar effects. Winer et al. [22] in a recent paper, carried out in a cohort of young women at their first intercourse demonstrated that consistent condom use reduces the risk of cervical HPV infection. It is worth noting that Winer's study is based on one year of observation whereas in our study, on average at baseline, 4.5 years had passed since the sexual debut.

At least two different explanations might account for these conflicting figures:

a) Information bias: information collected retrospectively could determine a misclassification bias. If this were the case, the relationship between condom use and HPV would move towards the null value.

b) The probability of developing HPV infection depends on from the time passed since the first intercourse and as protection is not absolute for condom users, in the long term, they also could be become infected. In particular it is worth mentioning that, as time passes, HPV infection tends to spread to areas not protected by condoms and HPV infections can still be transmitted through non-penetrative sexual contact or contact with areas of unprotected genital skin.

This study design will permit a prospective analysis, in a relatively short interval, of the role of different risk factors (i.e. sexual behaviour, smoking habits, condom use and so on) in the acquisition of new HPV infections (or with a different HPV type) for women negative at baseline. Furthermore, it might permit an evaluation of the role of these risk factors in the clearance of infections for women positive at baseline.

In the follow-up of this study we will analyze data from the yearly self-administered questionnaires regarding only the year to date. In this manner we aim to reduce reporting biases related to the large interval since the beginning of sexual activity and also with a temporal sequence of its progress.

## Competing interests

Financial competing interests

· In the past five years have you received reimbursements, fees, funding, or salary from an organization that may in any way gain or lose financially from the publication of this manuscript, either now or in the future? Is such an organization financing this manuscript (including the article-processing charge)?

NO

· Do you hold any stocks or shares in an organization that may in any way gain or lose financially from the publication of this manuscript, either now or in the future?

NO

· Do you hold or are you currently applying for any patents relating to the content of the manuscript? Have you received reimbursements, fees, funding, or salary from an organization that holds or has applied for patents relating to the content of the manuscript?

NO

· Do you have any other financial competing interests?

NO

The authors declare that they have not competing interests.

Non-financial competing interests

Are there any non-financial competing interests (political, personal, religious, ideological, academic, intellectual, commercial or any other) to declare in relation to this manuscript?

NO

The authors declare that they have not competing interests.

## Authors' contributions

MC conceived the study and participated in its design and coordination and helped to draft the manuscript. FC conceived the study and participated in its design and coordination and helped to draft the manuscript. MZ performed the statistical analysis and helped to draft the manuscript. LV performed the statistical analysis and helped to draft the manuscript. AI carried out the protocol organization in Florence. PC carried out the cytological analysis in Florence. LB performed the sampling collection in Florence. MF performed the colposcopic examination. FM carried out the protocol organization and the cytological analysis in Arezzo. PV carried out the protocol organization in Lucca. AS carried out the cytological analysis in Lucca. DB performed the sampling collection in Florence. CS carried out the molecular analysis, participated in the study coordination and helped to draft the manuscript. All authors read and approved the final manuscript.

## Pre-publication history

The pre-publication history for this paper can be accessed here:

http://www.biomedcentral.com/1471-2334/10/157/prepub
